# Topical cyclosporine A cationic ophthalmic emulsion in paediatric vernal keratoconjunctivitis: pooled analysis of randomised NOVATIVE and VEKTIS trials

**DOI:** 10.1038/s41433-022-02342-6

**Published:** 2022-12-23

**Authors:** Andrea Leonardi, Serge Doan, Pasquale Aragona, Mourad Amrane, Dahlia Ismail, Jesús Montero, János Németh, Dominique Bremond-Gignac

**Affiliations:** 1 Department of Neuroscience, Ophthalmology Unit, University of Padua, Padua, Italy; 2 Bichat Hospital and Foundation A. de Rothschild, Paris, France; 3 Department of Biomedical Sciences, Ophthalmology Clinic, University of Messina, Messina, Italy; 4 Santen SAS, Evry, France; 5 Universidad de Sevilla, Seville, Spain; 6 Department of Ophthalmology, Semmelweis University, Budapest, Hungary; 7 Department of Ophthalmology, University Hospital Necker Enfants Malades, APHP, Paris, France; 8 INSERM Unit 1138, T17 Paris Cité University, Paris, France

**Keywords:** Outcomes research, Paediatrics

## Abstract

**Background/Objectives:**

Cyclosporine A cationic ophthalmic emulsion (CsA CE) was evaluated in paediatric and adolescent patients with vernal keratoconjunctivitis (VKC) in the NOVATIVE (NCT00328653) and VEKTIS (NCT01751126) trials. The similarity of these studies permitted pooled assessment of the effect of CsA CE on corneal damage as well as safety and tolerability.

**Subjects/Methods:**

Pooled outcomes were assessed for the first 28 days of treatment. In NOVATIVE, 118 patients were randomised to 4 times daily (QID) CsA CE 0.05%, 0.1%, or vehicle eye drops. In VEKTIS, 169 patients were randomised to CsA CE 0.1% QID or twice daily (BID) or vehicle. For these analyses, treatment groups comprised: (1) pooled CsA CE 0.1% QID arms (high-dose; *n* = 96); (2) pooled CsA CE 0.05% QID arm from NOVATIVE and CsA CE 0.1% BID data from VEKTIS (low-dose; *n* = 93); and (3) pooled vehicle QID arms (vehicle; *n* = 98).

**Results:**

Changes from baseline to day 28 (mean ± standard deviation) in corneal fluorescein staining (CFS) scores for CsA CE high-dose, low-dose, and vehicle groups were −1.6 ± 1.47 (95% CI: −0.9, −0.1; *p* = 0.0124 vs vehicle), −1.7 ± 1.39 (95% CI: −1.1, −0.3; *p* = 0.0015 vs vehicle), and −1.0 ± 1.55, respectively. Adverse events (AEs) of any type were reported in 37.5%, 34.4%, and 37.8% of the high-dose, low-dose, and vehicle groups, respectively. Most were mild or moderate in severity.

**Conclusions:**

CsA CE significantly decreased corneal damage and was safe and well tolerated in patients with VKC. These data support CSA CE as a treatment option for the management of VKC.

## Introduction

Vernal keratoconjunctivitis (VKC) is a relatively rare form of ocular allergy that occurs most frequently in children and adolescents with seasonal recurrence [1, 2]. The signs and symptoms of VKC include itching and grittiness, photophobia, tearing, palpebral thickening, and associated pseudoptosis [3]. Vernal keratoconjunctivitis typically involves proliferative lesions in the bulbar and tarsal conjunctiva, including giant papillae formation, and may be characterised as tarsal, limbal, or mixed [4]. There are also alterations in goblet cell distribution, intercellular connections, and keratinisation [5]. Severe VKC is a serious condition that may result in sight-threatening complications such as central corneal scarring, corneal perforation, keratoconus, and hyperplasia of limbal tissues [6].

The pathology of VKC involves Th2 cells, eosinophils, dendritic cells, and mast cells, as well as a variety of chemokines, adhesion molecules, and cytokines [7–9]. A range of therapeutic approaches—including mast cell stabilisers, antihistamines, nonsteroidal anti-inflammatory drugs, topical or injected (supratarsal) corticosteroids, immunomodulators, and targeted immunotherapeutic agents—have been employed for the treatment of VKC [7]. The efficacy of these therapies appears to vary across the various signs and symptoms associated with VKC [10].

Although topical corticosteroids are acknowledged as effective anti-inflammatory agents in active ocular allergy, a key limitation of their use in severe VKC is the risk of glaucoma and/or cataract development with long-term use [11–14]. As a result, corticosteroids are not the preferred treatment choice for ocular allergy, and patients receiving corticosteroids should be monitored by an ophthalmologist [14].

Topical cyclosporine (CsA) has been shown to be effective for the treatment of VKC in multiple clinical studies and is recommended as a steroid-sparing therapy for this condition [14–17]. The utility of topical CsA for the treatment of VKC has been advanced by the development of cyclosporine A cationic ophthalmic emulsion (CsA CE) 0.1% (1 mg/mL) [18]. This cationic oil-in-water emulsion for topical ocular use remains on the ocular surface longer than conventional anionic CsA formulations, thereby optimising its bioavailability and therapeutic effects [19–21].

CsA CE 0.1% has been evaluated in patients with VKC in the multicentre, randomised, double-masked, vehicle-controlled phase II/lll NOVATIVE and the phase III VEKTIS trials, which assessed the efficacy and tolerability of CsA CE 0.1% eye drops for treatment of severe VKC in children and adolescents ≥4 years of age (NOVATIVE) and between 4 and <18 years of age (VEKTIS) [22, 23]. Few large-scale randomised controlled trials have been conducted in VKC and the multinational NOVATIVE and VEKTIS trials represent 2 of the largest prospective controlled studies of VKC treatment to date. Similarities in study designs, patient characteristics, and outcome measures permitted pooled analysis of safety and treatment effect on corneal damage as assessed by corneal fluorescein staining (CFS) scores in this large VKC cohort. Herein, we report pooled safety and CFS outcomes seen with CsA 0.1% treatment in the NOVATIVE and VEKTIS trials.

## Materials/subjects and methods

### Trial designs

#### NOVATIVE

**Fig. 1:**
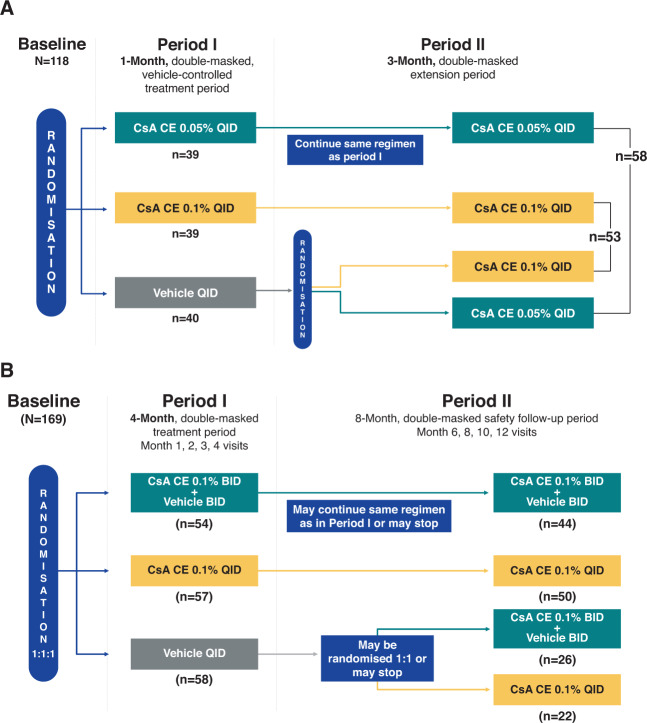
Study designs. Study designs and patient disposition, NOVATIVE (**A**) and VEKTIS (**B**) trials BID, twice daily; CsA CE, cyclosporine A cationic ophthalmic emulsion; QID, 4 times daily.

NOVATIVE (NCT00328653) was a phase II/III, multicentre, randomised, double-masked, parallel-group, double-ranging controlled trial. The study was conducted at 21 sites in 6 countries. The study included 2 treatment periods. In the first 4-week period, patients (*N* = 118) were randomised 1:1:1 to 4 times daily (QID) treatment with CsA CE 0.05%, CsA CE 0.1%, or vehicle eye drops. In the second, patients were administered CsA CE 0.05% or 0.1% QID or twice daily (BID) in both eyes for an additional 3 months (Fig. 1A). Randomisation numbers were allocated centrally by a third-party vendor (Orion Clinical Services Ltd., UK). Systemic or topical corticosteroids, mast cell stabilisers, and topical ocular antihistamines were discontinued prior to initiating study treatment and could not be used for the duration of the trial. Key inclusion and exclusion criteria are summarised in Table 1 and the primary efficacy endpoint was overall rating of subjective symptoms at week 4. A secondary endpoint was corneal fluorescein staining (CFS) scores as a measure of keratitis. The first patient entered the trial on 19 May 2006 and the date of the last visit of the last patient to complete the trial was 22 February 2007.

**Table 1:** Inclusion/exclusion criteria of the two clinical trials.

NOVATIVE	VEKTIS
Inclusion	Inclusion
• ≥4 years of age • Active VKC (acute or chronic) needing treatment • ≥2 following signs, in at least one eye: ○ Presence of giant papillae with a diameter ≥1 mm on the upper tarsal conjunctiva and superficial keratitis • ≥2 of the following ocular symptoms with a score >2 in at least one eye: burning/stinging, tearing, itching, pain, sticky eyelids, foreign body sensation, mucus discharge, and photophobia • Hyperaemia score ≥2 • Informed consent	• 4 to <18 years of age • Active severe VKC^*^ with severe keratitis^†^ • ≥1 recurrence of VKC during the previous year • Mean score of ≥60 mm on a 0–100-mm VAS for the 4 main VKC symptoms (photophobia, tearing, itching, and mucous discharge) • Informed consent
Exclusion	Exclusion
• Concomitant corneal ulcer of infectious origin • Active herpes • Disease that could possibly interfere with the interpretation of the trial results: active uveitis (defined by Tyndall score >0), previous history of ocular hypertension or glaucoma, or condition incompatible with the frequent assessments needed by the trial • History of malignancy or a recurrence in the last 5 years • Abnormality of nasolacrimal drainage apparatus • Concomitant disease not stabilised within 1 month before screening visit or incompatible with the trial • Severe systemic allergy requiring systemic treatment at trial entry • Female of childbearing potential • History of drug or alcohol addiction • Use of prohibited concomitant medications	• Ocular anomalies other than VKC affecting the ocular surface • Abnormalities of lid anatomic features, nasolacrimal drainage, or blinking function • Active ocular infection or history of ocular herpes, varicella zoster or vaccinia virus infection; or any ocular disease that would require topical ocular treatment during the study • Presence or history of severe systemic allergy • Topical or systemic corticosteroids within 1 week; topical CsA, tacrolimus, or sirolimus or any systemic immunosuppressive drug within 90 days before enrolment • Scraping of the vernal plaque within 1 month; or any other ocular surgery within 6 months prior to baseline visit

*CFS* corneal fluorescein staining, *CsA* cyclosporine, *VAS* visual analogue scale, *VKC* vernal keratoconjunctivitis.

*Grade 3 or 4 on the Bonini scale.

^†^CFS score of Grade 4 or 5 on the modified Oxford scale.

#### VEKTIS

VEKTIS (NCT01751126) was a phase III, multicentre, randomised, double-masked, vehicle-controlled, parallel-arm trial that included a 4-month efficacy/safety evaluation period and an 8-month double-masked safety follow-up period [22]. The study was conducted at 51 sites in 11 countries. Patients (*n* = 169) were randomised (1:1:1) to CsA CE 0.1% QID or BID or to vehicle (Fig. 1B). A computerised randomisation schema was used; randomisation was centralised using an Interactive Web Response System and was stratified by country. Following the 4-month evaluation period, CsA CE 0.1% patients who still presented with signs and symptoms of VKC were allowed to continue their assigned active treatment in a double-masked fashion. Patients in the vehicle group who continued to experience signs and symptoms of VKC after 4 months were allowed to switch to active treatment [22]. Rescue therapy with dexamethasone 0.1% eye drops was permitted and monitored throughout the study period. Efficacy was assessed using a composite efficacy score that encompassed keratitis (assessed by CFS and scored using the modified Oxford scale), need for rescue medication, and occurrence of corneal ulceration [18]. The CFS score was a secondary endpoint [18]. The first patient entered the trial on 29 Apr 2013 and the date of the last visit of the last patient to complete the trial was 01 Feb 2016.

Both studies were conducted in accordance with the Declaration of Helsinki and Good Clinical Practice guidelines. Independent ethics committees and regulatory agencies (as appropriate) approved the study protocol before the trial was initiated (Supplementary Table 1). A parent or legal guardian for each patient provided written informed consent, and the patient provided assent when possible [22, Data on File].

### Pooled analysis of CFS scores and safety data

Efficacy results for CFS and safety data from the vehicle-controlled periods of both studies were pooled into the following treatment arms:

- Pooled low-dose group (*n* = 93): CsA CE 0.05% QID arm from NOVATIVE and CsA CE 0.1% BID arm from VEKTIS
- Pooled high-dose group (*n* = 96): CsA CE 0.1% QID arms from both trials
- Pooled vehicle group (*n* = 98): Pooled vehicle QID arms from both trials

Safety assessments for both studies included laboratory data, best-corrected distance visual acuity (BCDVA), and systemic and ocular adverse event (AE) reporting, including intraocular pressure (IOP) assessment. Change from baseline in CFS scores by dose group was assessed using an ANCOVA model including baseline CFS score and Study as covariates. Missing data at day 28 was replaced by the last post-treatment observation available (last observation carried forward method).

## Results

### Patients

#### Disposition

In the NOVATIVE trial, 118 patients were enrolled and randomised. The safety and efficacy analysis sets (full analysis set) included all patients who received treatment. At the end of the first period (day 28), 111 patients (94.1%) remained in the trial: 4 patients withdrew from the vehicle group, and 3 from the CsA CE 0.1% group. The most common reasons for discontinuation among patients who withdrew were worsening of disease (42.9%, all in vehicle group) and patient decision/withdrawal of consent (28.6%, all in CsA CE 0.1% group).

In the VEKTIS trial, a total of 169 patients were randomised to study treatment. Of these, 143 (84.6%) completed the 4-month treatment period. Of those who did not complete treatment period, 9 were in the vehicle group, 11 were in the CsA CE 0.1% BID group, and 6 were in the QID group. The most frequent reasons for discontinuation among withdrawn patients were lack of efficacy (42.3%) and patient decision unrelated to an AE (26.9%) [18].

#### Pooled baseline characteristics

Baseline characteristics, including demographic data, medical history, and medication history, were comparable among treatment groups in both studies (Table 2). Baseline data for the pooled analysis also indicated comparable demographic and clinical characteristics across the 3 treatment groups.

**Table 2:** Patient characteristics.

	High-Dose Regimen* (*n* = 96)	Low-Dose Regimen^†^ (*n* = 93)	Vehicle (*n* = 98)	Total (*N* = 287)
Age^‡^				
Mean, years (SD)	9.2 (3.4)	9.1 (3.2)	8.7 (2.9)	9.0 (3.2)
4–11 years, *n* (%)	73 (76.0)	71 (76.3)	79 (80.6)	223 (77.7)
12–18 years, *n* (%)	22 (22.9)	22 (23.7)	19 (19.4)	63 (22.0)
Sex, *n* (%)				
Male	78 (81.3)	75 (80.6)	75 (76.5)	228 (79.4)
Form of VKC, *n* (%)				
Limbal	8 (8.3)	2 (2.2)	7 (7.1)	17 (5.9)
Tarsal	23 (24.0)	20 (21.5)	29 (29.6)	72 (25.1)
Both	65 (67.7)	71 (76.3)	62 (63.3)	198 (69.0)
Type of VKC, *n* (%)				
Seasonal	38 (39.6)	35 (37.6)	30 (30.6)	103 (35.9)
Perennial	58 (60.4)	58 (62.4)	68 (69.4)	184 (64.1)
Mean time since diagnosis, years (SD)	3.8 (2.6)	3.5 (2.4)	3.3 (2.4)	3.5 (2.5)
CFS at baseline, *n* (%)				
Grade ≤2	15 (15.6)	10 (10.8)	11 (11.2)	36 (12.5)
Grade 3	10 (10.4)	14 (15.1)	14 (14.3)	38 (13.2)
Grade 4	54 (56.3)	59 (63.4)	63 (64.3)	176 (61.3)
Grade 5	17 (17.7)	10 (10.8)	10 (10.2)	37 (12.9)

*BID* twice daily, *CFS* corneal fluorescein staining, *CsA CE* cyclosporine A cationic ophthalmic emulsion, *QID* 4 times daily, *SD* standard deviation, *VKC* vernal keratoconjunctivitis.

*CsA CE 0.1% QID arms from both trials.

^†^CsA CE 0.05% QID arm from NOVATIVE and CsA CE 0.1% BID data from VEKTIS.

^‡^Day and month of birth were missing for 1 patient in the NOVATIVE study; they were replaced by the 1st of July for the calculation of age (11 years).

### Efficacy

#### NOVATIVE

Change from baseline in CFS scores indicated that treatment with CsA 0.05% and 0.1% produced significant improvements from baseline in corneal staining compared with vehicle (*p* = 0.0135 for 0.05% and *p* = 0.0027 for 0.1%; results for worse eye). Overall ratings of subjective symptoms of VKC indicated improvements from baseline, but no significant differences between the CsA CE 0.05% QID or 0.1% QID dose groups were observed compared with vehicle (*p* = 0.2699 and *p* = 0.2719, respectively; Mantel-Haenszel Chi-square test). Overall worsening of subjective symptoms was higher in the vehicle group (17.5%) versus the CsA CE 0.1% (10.3%) and CsA CE 0.05% (2.6%) groups. Overall rating of objective signs of VKC at day 28 indicated significant superiority of both 0.05% and 0.1% CsA CE treatments over vehicle (*p* = 0.0386 and *p* = 0.0208, respectively; Mantel-Haenszel Chi-square test).

#### VEKTIS

The composite efficacy score increased significantly over the treatment period. The difference in the least-squares (LS) mean for both the CsA CE 0.1% QID (0.76; 95% CI: 0.26, 1.27) and BID group (0.67; 95% CI: 0.16, −1.18) was significant compared with vehicle (*p* = 0.007 and *p* = 0.010, respectively). The primary driver of improvement in the composite score was the change from baseline in CFS score (QID: LS mean 0.52; 95% CI: 0.11, 0.94; BID: LS mean 0.53; 95% CI: 0.11, 0.94, *p* = 0.014 vs vehicle for both groups), which accounted for 70.3% of the treatment effect in the QID group and 77.6% in the BID group. Compared with vehicle-treated patients, at 4 months patients in the CsA CE QID group reported statistically significant reductions in photophobia (LS Mean −20.86; 95% CI: −32.36, −9.36; *p* < 0.001), tearing (LS Mean −15.61; 95% CI: −26.25, −4.96; *p* = 0.009), itching (LS Mean −19.46; 95% CI: −30.58, −8.33; *p* = 0.001), and mucous discharge (LS Mean −17.96; 95% CI: −29.57, −6.34; *p* = 0.005), as well as significant improvement in QoL as assessed with the Quality of Life in Children with Vernal Keratoconjunctivitis (QUICK) [24] questionnaire symptoms domain (LS Mean −8.77; 95% CI: −16.40, −1.13; *p* = 0.049) and daily activities domain (LS Mean −10.33; 95% CI: −17.46, −3.20; *p* = 0.009). There was also less use of rescue medication in the active treatment groups compared to vehicle (QID LS Mean 0.22; 95% CI: 0.068, 0.372; *p* = 0.010; BID LS Mean 0.15; 95% CI: −0.00, 0.30; *p* = 0.055) [18].

##### Pooled CFS scores

**Fig. 2:**
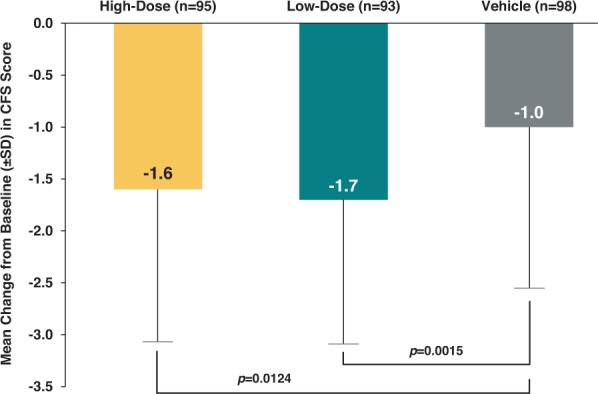
Mean change from baseline to day 28 in CFS score (pooled results). Based on ANCOVA model including baseline CFS score and Study as covariates. Missing data at day 28 has been replaced by the last post-treatment observation available (LOCF method). Low-dose regimen: CsA CE 0.1% BID or 0.05% QID, High-dose regimen: CsA CE 0.1% QID. ANCOVA analysis of covariance, BID twice daily, CFS corneal fluorescein staining, CsA CE cyclosporine A cationic ophthalmic emulsion, LOCF last observation carried forward, QID 4 times daily, SD standard deviation.

Pooled results for CFS scores at day 28 indicated significant improvements for the CsA CE high- and low-dose groups compared with vehicle (Fig. 2). Mean change from baseline (± standard deviation) in pooled CFS scores were −1.6 ± 1.47, −1.7 ± 1.39, and −1.0 ± 1.55, for the CsA CE high-dose, CsA CE low-dose, and vehicle groups respectively (*p* = 0.0124 for high-dose and *p* = 0.0015 for low-dose vs vehicle) [25].

### Safety and tolerability

Adverse events of any type were reported in 37.5%, 34.4%, and 37.8% of patients in the CsA CE high-dose, CsA CE low-dose, and vehicle groups, respectively (Table 3). The respective values for drug-related AEs were 21.9%, 17.2%, and 17.3%, respectively. Most AEs, including drug-related AEs, were mild or moderate in severity. No deaths occurred in either trial. Serious AEs occurred in 2, 1, and 0 patients in CsA CE high-dose, CsA CE low-dose, and vehicle groups, respectively. Discontinuations due to treatment-emergent AEs (TEAEs) were recorded in 5.2% of patients in the CsA CE high-dose group, no patients in the low-dose group, and 10.2% of patients in the vehicle group [26].

**Table 3:** Treatment-emergent adverse events (TEAEs) and treatment-related ocular TEAEs.

	High-Dose Regimen, *n* (%) (*n* = 96)	Low-Dose Regimen, *n* (%) (*n* = 93)	Vehicle, *n* (%) (*n* = 98)
TEAEs*			
All TEAEs	36 (37.5)	32 (34.4)	37 (37.8)
Drug-related TEAEs	21 (21.9)	16 (17.2)	17 (17.3)
Severity of TEAEs			
Mild	16 (16.7)	24 (25.8)	13 (13.3)
Moderate	14 (14.6)	2 (2.2)	18 (18.4)
Severe	6 (6.3)	6 (6.5)	6 (6.1)
Severity of drug-related TEAEs			
Mild	11 (11.5)	13 (14.0)	5 (5.1)
Moderate	8 (8.3)	1 (1.1)	9 (9.2)
Severe	2 (2.1)	2 (2.2)	3 (3.1)
Death	0	0	0
SAE	2 (2.1)	1 (1.1)	0
Drug-related SAE	0	0	0
Discontinuation due to TEAEs	5 (5.2)	0	10 (10.2)
Discontinuation due to drug-related TEAEs	3 (3.1)	0	5 (5.1)
Treatment-related ocular TEAEs (System Organ Class Preferred Term)**			
General disorders & administration site conditions	13 (13.5)	16 (17.2)	6 (6.1)
Instillation site pain	9 (9.4)	7 (7.5)	4 (4.1)
Instillation site pruritus	5 (5.2)	7 (7.5)	3 (3.1)
Instillation site erythema	0	1 (1.1)	2 (2.0)
Drug intolerance	0	2 (2.2)	0
Application site discharge	0	0	1 (1.0)
Application site swelling	0	1 (1.1)	0
Eye disorders	10 (10.4)	2 (2.2)	9 (9.2)
Ocular hyperemia	3 (3.1)	2 (2.2)	1 (1.0)
Visual acuity reduced	3 (3.1)	0	2 (2.0)
Allergic keratitis	1 (1.0)	0	1 (1.0)
Ulcerative keratitis	0	0	2 (2.0)
Blepharospasm	1 (1.0)	0	0
Cataract subcapsular	0	0	1 (1.0)
Corneal leukoma	0	0	1 (1.0)
Eye irritation	1 (1.0)	0	0
Eye pain	1 (1.0)	0	0
Eyelid erosion	1 (1.0)	0	0
Eyelid oedema	0	0	1 (1.0)
Investigations	0	0	1 (1.0)
Increased IOP	0	0	1 (1.0)

*AE* adverse event, *IOP* intraocular pressure, *Med DRA* medical dictionary for regulatory activities, *SAE* serious adverse event, *TEAE* treatment-emergent adverse event.

*A patient was counted only once in his/her maximal severity.

**If a subject had more than one AE within the preferred term, he or she was counted only once; categories derived from Med DRA Version 19.0.

#### Ocular adverse events

Ocular AEs followed the same pattern as overall AEs. Ocular TEAEs were reported for 29.2% of patients in the CsA CE high-dose group, 24.7% of the low-dose group, and 26.5% of those in the vehicle group. Most were mild or moderate in severity. There was 1 serious ocular AE of severe ulcerative keratitis in the CsA CE high-dose group that was considered unrelated to study treatment and that resolved with sequelae of corneal leukoma. Discontinuations due to ocular TEAEs were reported for 5.2% of patients in the CsA CE high-dose group, no patients in the low-dose group, and 8.2% of patients in the vehicle group.

#### Drug-related ocular TEAEs

Drug-related ocular TEAEs were reported for 20.8% of patients in the CsA CE high-dose group, 17.2% of those in the low-dose group, and 14.3% of those in the vehicle group. No serious ocular AEs were reported as related to study treatment. Discontinuation due to drug-related ocular TEAEs occurred in 3.1% of patients in the CsA CE high-dose group, no patients in the low-dose group, and 3.1% in the vehicle group. Individual drug-related ocular TEAEs are summarised in Table 3; the most frequent were instillation site pain (9.4%, 7.5%, and 4.1% in the high-dose, low-dose, and vehicle groups) and instillation site pruritus (5.2%, 7.5%, and 3.1%, respectively). A single case of elevated IOP was reported in a patient in the vehicle group, which resolved without sequelae within 3 months.

##### Systemic/non-ocular adverse events

Non-ocular drug-related AEs were infrequent, indicating support for minimal systemic exposure to CsA with topical CsA CE. One patient in the CsA CE high-dose group reported rhinorrhoea and a second patient reported headache. One patient each in the vehicle group reported throat tightness, rash, or urticaria.

## Discussion

Pooled analysis of data from the NOVATIVE and VEKTIS studies indicate that both regimens were significantly more effective than vehicle in improving keratitis as measured by CFS scores in patients with VKC. Improvements were observed by 28 days after treatment initiation. Although other efficacy endpoints were evaluated in the NOVATIVE and VEKTIS trials, only CFS scores were included in these pooled analyses because the approaches to assessment of corneal staining were comparable in both the trials. The CFS score is particularly important in the assessment of patients with VKC because it directly assesses corneal involvement and the risks for complications such as corneal ulceration and scarring that may result in impaired vision [27]. The inclusion of patients from the VEKTIS trial who received rescue medication could be viewed as a potential confounder and deserves further analysis.

Pooled analysis of data from the NOVATIVE and VEKTIS studies also demonstrated that high- and low- dose regimens of CsA CE were safe and well tolerated. The most frequently reported AEs were usually non-serious, transitory, and mild or moderate in severity. In addition, only 2 systemic AEs were reported, 1 patient each with headache or rhinorrhoea. The favourable safety profile for CsA CE demonstrated by this pooled analysis of 4-week safety data is supported by longer term follow-up of patients receiving CsA CE for up to 12 months in the VEKTIS trial [22]. The long-term results from the VEKTIS study indicate no increase in the rate of AEs compared with shorter follow-up periods. During long-term follow-up, local AEs generally occurred early during CsA CE treatment and declined with continued use of the drug. Also, there was a low rate of discontinuation due to TEAEs.

No clinically significant changes in visual acuity, IOP, or slit lamp examination findings were reported with long-term use of CsA CE [22]. In addition, systemic absorption of CsA was negligible and below the upper limit of quantification (5 ng/mL). These results support the use of CsA CE as a safe alternative to topical corticosteroids for the treatment of VKC. As noted previously, steroids have been shown to be effective for the treatment of moderate-to-severe VKC, but should only be employed for short courses of therapy due to an increased risk for severe AEs, including glaucoma, cataracts, and secondary infections of the cornea [3, 28].

The pooled safety analysis for CsA CE indicates that the high-dose regimen is as safe and tolerable as the lower dose. This result, coupled with its long-term efficacy, prompted selection of 1 drop of CsA CE 0.1% instilled 4 times per day as the recommended dosing regimen [22, 23].

The limitations of pooled analyses are well documented, particularly when pooling studies with different populations, treatment practices, or designs [29]. While the entry criteria for the NOVATIVE and VEKTIS trials were different, the overall study designs and characteristics of the patients enrolled in the 2 studies were similar (Table 1, Fig. 1).

Combining results from different treatment regimens might also be expected to increase the heterogeneity of results and to decrease the probability of detecting a significant difference compared with control [30]. This is particularly relevant with regard to the pooling of results from patients administered CsA CE 0.05% QID and 0.1% BID into a single low-dose group. It is therefore noteworthy that significant efficacy was demonstrated for both CsA CE groups compared with the vehicle control. Although it might be argued that these trials lacked a “true” placebo comparator because any topical agent may provide benefit by diluting inflammatory mediators and allergens; this limitation would result in underestimation of the clinical benefit of CsA CE compared with control treatment. In addition, pooling data for evaluation of CsA CE safety can provide confidence in the consistency of the safety profile across a wider range of patient types and dosing regimens [29].

The results from these pooled analyses support the conclusion that CsA CE is safe, well tolerated, and significantly improves keratitis in patients with active VKC. Combined with previously published data on the efficacy of CsA CE in paediatric VKC [18, 22], these findings further support the use of CsA CE as a treatment option for the management of children and adolescents with moderate-to-severe VKC.

## Summary Table

### What was known before

- Cyclosporine has been used off label for the treatment of vernal keratoconjunctivitis (VKC) since it was first approved in 1983.
- Cyclosporine A cationic ophthalmic emulsion (CsA CE) is a unique formulation of cyclosporine that is specifically approved for use in patients with VKC.

### What this study adds

- This analysis of pooled data from 2 pivotal randomised trials of CsA CE demonstrated that high- and low-dose regimens of CsA CE were safe, well tolerated, and significantly improved keratitis as measured by corneal fluorescein staining scores in patients with VKC.
- These results support the use of CsA CE in children and adolescents as a safe alternative to topical corticosteroids for the treatment of VKC.

## Supplementary information

Supplemental Table 1 Ethics Committees

## Data Availability

The datasets analysed in the manuscript are available from the corresponding author on reasonable request.
