# Supplementary material for: Topical cyclosporine A cationic ophthalmic emulsion in paediatric vernal keratoconjunctivitis: pooled analysis of randomised NOVATIVE and VEKTIS trials
Source: Eye (Lond). 2022 Dec 23;37(11):2320–6. doi: 10.1038/s41433-022-02342-6 (PMC10366270; doi:10.1038/s41433-022-02342-6)
Supplement: Supplementary file 1 — Supplemental Table 1 Ethics Committees [file 41433_2022_2342_MOESM1_ESM.docx]

Supplemental Table 1: Ethics Committees

| **Croatia** | | |
| --- | --- | --- |
| Central Ethics Committee  Ksaverska cesta 4  Zagreb, Croatia 10000 |  |  |
| **France** | | |
| Centre Hospitalier Universitaire Niveau 3 – porte 508 Avenue de la Côte de Nacre CAEN cedex 9 France 14033 | Monsieur Guirimand  Hôpital Ambroise Paré  Laboratoire d’Anatomo-Pathologie  9 av. Charles de Gaulle  Boulogne Billancourt  France 92100 |  |
| **Germany** | | |
| Ethik-Kommission bei der Landesärztekammer Rheinland-Pfalz Deutschhausplatz 3 Mainz, Germany 15116 | Ethikkommission der Med. Fakultät der HHU Düsseldorf Kinderklinik Geb. 13.41 Moorenstr. 5 Düsseldorf, Germany 40225 |  |
| **Greece** | | |
| National Ethics Committee  284 Mesogeion Avenue  Cholargos  Athens, Greece 15562 |  |  |
| **Hungary** | | |
| Ethics Committee for Clinical Pharmacology of the Medical Research Council (Egészségügyi Tudományos Tanács Klinikai Farmakológiai Etikai Bizottsága, ETT-KFEB) Arany János utca 6-8 Budapest Hungary H-1051 |  |  |
| **India** | | |
| Vision Research Foundation No:18 College Road Chennai TamilNadu  India 600006 | Institutional Ethics Committee Andhra Medical College Opp Bulliya College Resapuvanipalem Vishakapatnam Andhra Pradesh  India 530002 | Sparsh Hospitals Ethics Committee for Human Research A-407 Sahid nagar Bhubaneshwar  Orissa  India 751007 |
| Institutional Review Board Acharya Donde Marg  Parel  Maharashtra  Mumbai, India 400012 | Institutional Ethics Committee  Maulana Azad Medical College  Bahadur Shah Zafar Marg  New Delhi -2, India | Institutional Ethics Committee  King George’s Medical University  Chowk  Lucknow, India |
| **Israel** | | |
| Helsinki Committee Kaplan Medical Center  Pasternak St. p.o.b. 1  Rehovot  Israel 76100 | The Medical Research, Infrastructure, and Health Services fund of the Tel Aviv Medical Center  6 Weizmann Street  Tel-Aviv  Israel 64239 | Helsinki Committee Rabin Medical Center  39 Jabotinsky St.  Petah-Tikva  Israel 49100 |
| Helsinki Committee Soroka University Medical Center (affiliated with Ben Gurion University)  p.o.b. 151  Be’er-Sheva, Israel 84101 | Helsinki Committee Hadassah Medical Organization  Kiryat Hadassah p.o.b. 12000  Jerusalem, Israel 91120 | Meir Hospital – Institutional Review Board Committee  Sapir Medical Centre  Kfar Saba Sackler Faculty of Medicine  Tel-Aviv University, Israel |
| Institutional Review Board Committee  Hadassah University Hospital  Dept. Of Ophthalmology  Jerusalem, Israel | Institutional Review Board Committee  Soroka University Medical Centre  Beer Sheva, Israel 84101 | Institutional Review Board Committee  The Chaim Sheba Medical Centre (affiliated to Tel-Aviv University Sackler School of Medicine)  Tel-Hashomer, Israel 52621 |
| **Italy** | | |
| Comitato Etico Indipendente dell’ Azienda Ospedaliero-Universitaria  Policlinico S. Orsola-Malpighi di Bologna Via Albertoni, 15  Bologna, Italy 40138 | Comitato Etico Locale per la Sperimentazione dei Farmaci Dell’Azienda Ospedaliero-Universitaria Anna Mayer di Firenze  Viale Pieraccini, 24  Firenze, Italy 50139 | Comitato Etico per la Sperimentazione dell’Azienda Ospedaliera di Padova  Via Giustiniani, 1  Padova, Italy 35128 |
| Comitato per la Sperimentazione Clinica dei Midicinali Dell’Azienda Ospedaliero Universitaria Pisana di Pisa  Via Roma, 67  Pisa, Italy 56126 | Comitato Etico Scientifico dell’Azienda Ospedaliera Universitaria Poloclinico Gaetano Martino di Messina  Via Consolare Valeria  Messina, Italy 98125 | Comitato Etico ASL4 Chiavarese  Via GB Ghio 9  Chiavari  Italy 16043 |
| Comitato Etico  Universita Campus Bio Medico di Roma  Via Emilio Longoni, 83  Roma, Italy 00155 | Direzione Generale  Comitato Etico per la Sperimentazione  c/o Direzione Generale  via Giustiniani 1  Azienda Ospedaliera di Padova  Padova, Italy 35128 |  |

| **Morocco** | | |
| --- | --- | --- |
| Comité d’Ethique et de Déontologie  Université Mohammed V – Souissi  Faculté de Médecine et de Pharmacie  BP 6203 – Rabat, Morocco |  |  |
| **Portugal** | | |
| Comissão de Ética para a Investigação Clinica  Av. do Brasil, 53 – Pav. 17-A  Lisboa, Portugal 1749-004 |  |  |
| **Spain** | | |
| H. Torrevieja  Hospital de Torrevieja  Unidad de Investigación Clínica, Planta  1 Pasillo de Gerencia  Ctra. De Torrevieja a San Miguel de Salinas Cv-95  Pda. La Ceñuela s/n  Torrevieja (Alicante)  Spain 03186 | Euskadi Departamento de Sanidad  Gobierno Vasco  Edificio Lakua I, 4^a^ Planta  c/Donostia-San Sebastian  Spain 20014 | Consorcio Hospital General Universitario de Valencia  Hospital General Universitario de Valencia  Avda/Tres Cruces  Spain 46014 |
| Oftalmología Médica  Bifurcación Pío Baroja-General Avilés  Valencia, Spain 46015 | Autonómico de Andalucía  Consejería de Salud.  Avda Innovación, s/n.  Edificio Arena 1  Sevilla, Spain 41020 | Vissum Corporación  Oftalmológica  Vissum Corporación Oftalmológica, S.L.  C/ Cabañal, 1 - 4ª pl  Alicante, Spain 03016 |
| Euskadi  Departamento de Sanidad Gobierno Vasco  Edificio Lakua I, 4ª Planta.  c/ Donostia-San Sebastian  Vitoria, Spain 01010 | Instituto de Investigación Hospital, Unidad Administrativa  Area de Gestión de Proyectos – Gestión de la Investigación Instituto de Investigación  Hospital Centro de Actividades Ambulatorias  Avda de Córdob  Madrid, Spain 28041 | Galicia  Edificio Administrativo de San Lázaro  15703 Santiago de Compostela  Spain |
| Hospital Universitario La Paz  Paseo de la Castellana  Hospital General  Madrid, Spain 28046 | Hospital Infantil Universitario  Niño Jesús  Avenida Menendez y Pelayo 65  Madrid, Spain 28009 |  |
| Instituto Oftalmológico de Alicante  C/Cabañal, 1  Alicante 03016, Spain | Hospital Clinico San Carlos de Madrid  CIUDAD Universitaria  Madrid, Spain 28040 | Hospital Universitario de la Princesa  C/ Diego de León, 62  Madrid, Spain 28060 |
| **United States** | | |
| Copernicus Group Institutional Review Board  One Triangle Drive, Suite 100  Durham, North Carolina, USA 27709 | University of California, Irvine Office of Research Administration  5171 California Ave., Suite 150  Irvine, California, USA 92697 | University of Miami Institutional Review Board  1500 NW 12th Avenue  Suite 1002  Miami, Florida USA 33136 |
| **Turkey** | | |
| TC Sağlik Bakanliği  Iiaç ve Eczacilik Genel Müdürlüğü  Kalite Kontrol Dairesi Bşk.  Mithatpaşa Cad. No: 3  Sihhiye  Ankara, Turkey 06410 |  |  |
